# Exosomal cargos modulate autophagy in recipient cells via different signaling pathways

**DOI:** 10.1186/s13578-020-00455-7

**Published:** 2020-08-01

**Authors:** Mehdi Hassanpour, Aysa Rezabakhsh, Jafar Rezaie, Mohammad Nouri, Reza Rahbarghazi

**Affiliations:** 1 Stem Cell Research Center, Tabriz University of Medical Sciences, Imam Reza St., Golgasht St., Tabriz, 5166614756 Iran; 2 Department of Clinical Biochemistry and Laboratory Medicine, Tabriz University of Medical Sciences, Tabriz, Iran; 3 Stem Cell and Regenerative Medicine Institute, Tabriz University of Medical Sciences, Tabriz, Iran; 4 Cardiovascular Research Center, Tabriz University of Medical Sciences, Tabriz, Iran; 5 Solid Tumor Research Center, Cellular and Molecular Medicine Research Institute, Urmia University of Medical Sciences, P.O. Box: 1138, Shafa St, Ershad Blvd., Urmia, 57147 Iran; 6 Department of Applied Cell Sciences, Faculty of Advanced Medical Sciences, Tabriz University of Medical Sciences, Tabriz, Iran

**Keywords:** Exosomes, Extracellular vesicles, Autophagy, Exosome-induced autophagy

## Abstract

Vesicular system of mammalian cells is composed of two intracellular and extracellular vesicles systems, which contributes to the intra/intercellular communication and cellular homeostasis. These systems mediate transferring of biological molecules like proteins, nucleic acids, and lipids inside the cytoplasm, and between the cells. By the present study, authors describe molecular crosslink between exosome biogenesis and autophagy and take a certain focus on the autophagic cargos of exosomes and signaling pathways involved in exosome-induced autophagy in target cells and vice versa. Autophagy the generation of double-phospholipid vesicles, is a process that engulfs damaged proteins and organelles, share molecular similarity and function synergy with exosomes biogenesis for degradation or exocytosis of certain cargo. Exosomes, the smallest subtype of extracellular vesicles, originating from the membrane of the multivesicular body located inside cells demonstrate key roles in the intracellular and intercellular communication. Growing evidence demonstrates the interaction between exosome biogenesis and autophagy both at intertwined molecular pathways and crossbred vesicles known as amphisomes. Crosstalk between exosome biogenesis and autophagy contributes to maintain cellular homeostasis under external and internal stresses. Moreover, these processes can modulate each other via different signaling pathways. Exosomes contain autophagic cargos that induce autophagy via the cascade of molecular events in target cells, which called here exosome-induced autophagy. Taken together, crosstalk between exosome biogenesis and autophagy plays pivotal roles in cell homeostasis. Shedding light on the interaction between endomembrane systems may promote our knowledge about the relation between exosome and autophagy pathways in lysosome-related disorders against treatments; proposing a theoretical approach for therapy.

## Background

The endomembrane system of the mammalian cells encompasses the membranes and organelles that collaborate to maintain homeostasis through modifying, sorting, and transferring lipids, nucleic acids, and proteins [1, 2]. Various organelles including the nuclear envelope, endoplasmic reticulum, Golgi apparatus, and lysosomes participate to mediate different essential processes such as importing and exporting of different bio-molecules [1, 2]. Autophagy, a self-degrading process, has been considered as a dynamic process that plays pivotal roles in homeostasis of cells, especially in stressful conditions [3]. Unwanted/damaged molecules and organelles are degraded by the autophagic activity of cells, therefore, cells remain safe against stress [3]. Energy balance and ATP content of cell regulate autophagy flux, therefore, these factors could ignite autophagic switch on/off based on cell status [4].

Autophagy may link with other endomembrane systems as well as signaling pathways to regulate endocytosis, exocytosis, and even hydrolysis of bio-molecules [5, 6]. The ability of extracellular vesicles (EVs), especially those derived from endosomal system, exosomes, to cooperate with autophagy flux for preserving cellular homeostasis has recently been reported [7]. Exosomes are known as the smallest EVs that originate from late endosome (multivesicular body (MVB)) located at the cytoplasm ([8] {Jabbari, 2019#135)}. These vesicles released from most cells mediate intercellular communication by transferring bio-active molecules such as various proteins, lipids, RNAs and even DNA strands [9]. Besides, exosomes may participate to expel, degrade, and recycle of biomolecules, which may support the idea that exosome and autophagy pathways work together to promote cell survival [10, 11]. Through constant recycling of bio-molecules, cells achieve their metabolic demand and refurbish essential organelles, which support proliferation, growth, differentiation, and the management of physiological offers [12]. Confirmed that, in physiological conditions, autophagy facilitates cellular metabolism and homeostasis, however, it also mediates the pathogenesis of several diseases [13, 14]. Similarly, exosome biogenesis plays pivotal roles in normal condition and progression of different diseases. In light of recent studies, there is now evidence that both processes may synergically and alternatively act to support cells and the constituent of these endomembrane systems is structurally and functionally interlocked [15]. Outlining these complex networks may expand our knowledge about underlying mechanisms involved in vesicular trafficking, the fate of cargos of vesicles, the key roles of these vesicles in both intracellular and intercellular communication, and progression of lysosomal diseases. Here, we discuss the recent progress on the crosslink between exosome biogenesis and autophagy pathways; and also describe signaling pathways involved in mediating exosome-induced autophagy and vice versa.

## Autophagy

**Fig. 1:**
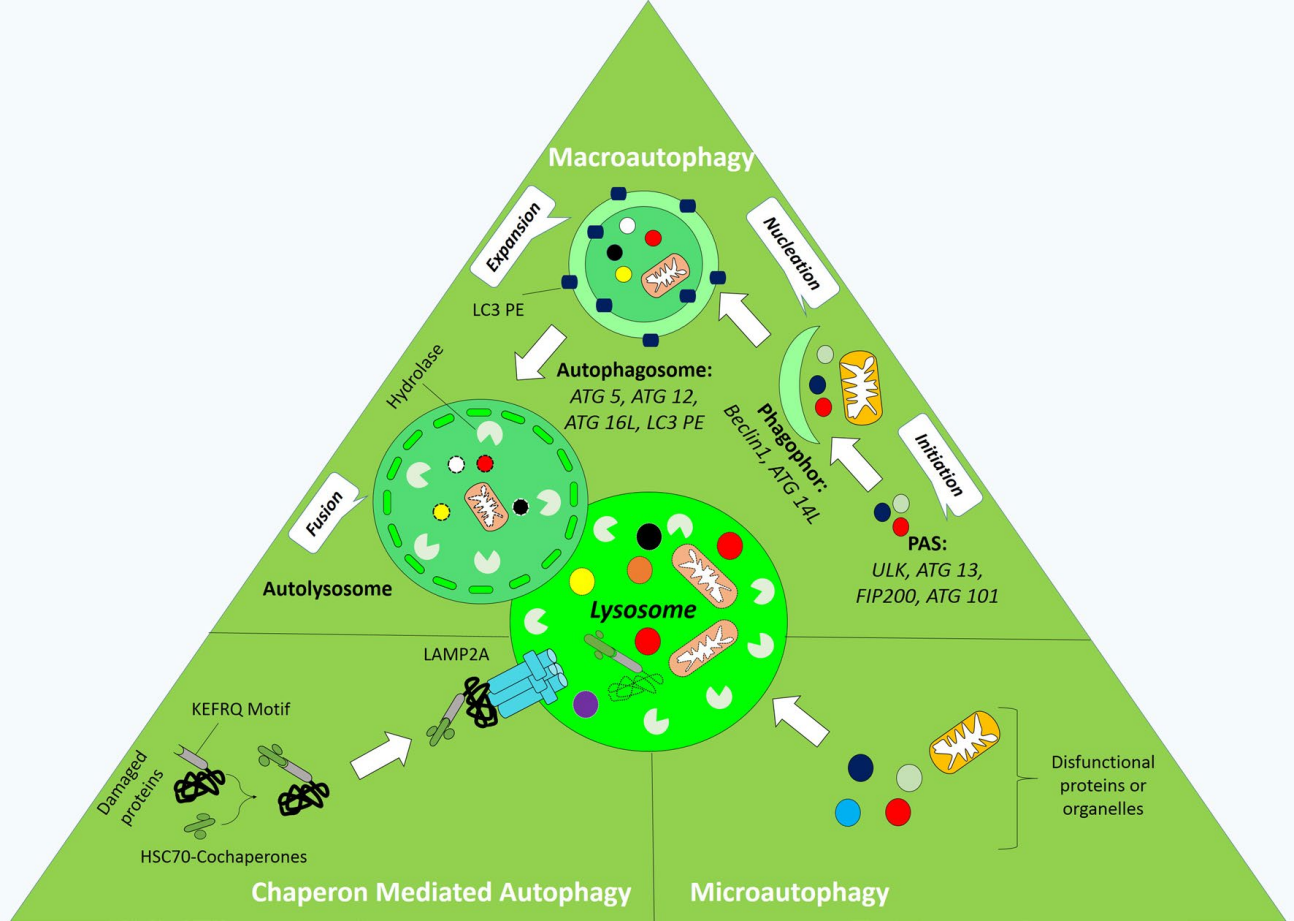
The autophagy flux. A diagram showing the autophagy and main regulatory molecules of autophagy pathway is presented. Three forms of autophagy may arise in cells; microautophagy, chaperone-mediated autophagy, and macroautophagy. Microautophagy is the procedure that impaired biomaterials directly sorted into lysosomes. In the chaperone-mediated autophagy, HSC70 classifies proteins containing specific motifs (KFERQ) and directs them into lysosome via interaction with LAMP2A molecules sited on lysosome membrane. Macroautophagy (autophagy) facilitates the lysosomal degradation of impaired proteins and organelles through four steps including initiation, nucleation, expansion, and finally fusion the autophagosome with lysosomes. Several proteins such as ULK, ATG13, FIP200, Beclin-1, ARG101, ATG5, ATG14L, ATG16L, LC3, and PE, in several steps, facilitate the development of autophagosome

protein metabolism (degradation and synthesis) is fundamental to maintain cellular homeostasis [16]. The interplay between the ubiquitin–proteasome system and autophagy pathway enables cells to recycle/deport intracellular unwanted/impaired proteins and organelles [3]. Autophagy is a complex process that mediates the degradation of unwanted proteins and dysfunctional organelles through fusion with lysosomes or by expeling them outside of the cell in such condition [17]. According to literature [18, 19] (Fig. 1), three types of autophagy have been documented; (I) Macroautophagy: a dual lipid membrane, autophagosome, surrounds the exhausted materials and fuses with lysosomes to form autophagolysosomes; (II) Microautophagy enters directly substances into the lysosomes through the intrusion of self-membrane and finally (III) Chaperone-mediated autophagy (CMA) degrades target molecules by engaging specific motifs (KFERQ) targeted by Heat shock cognate 71 kDa protein (HSC70) complex and then adhere to lysosomes via lysosomal-associated membrane protein type 2A (LAMP2A) [20]. Several stimuli facilitate autophagy flux/returns. For instance, reactive oxygen species (ROS), hypoxia, and starvation could contribute to autophagy flux [21]. The nutrient availability has been demonstrated to mediate autophagy flux through the organized targeting of rapamycin (mTOR) signaling pathway. In cells that are rich in nutrients and growth factors, the mTOR complex 1 (mTORC1) down-regulates the autophagy by phosphorylation and inhibition of the autophagy-initiating kinase Unc-51-like kinase 1(ULK1) and ATG13. On the contrary, the nutrient starvation inhibits the mTORC1, accordingly induces autophagy flux to save energy [22]. As shown in Fig. 1, the autophagy process comprises multiple steps such as initiation, elongation, expansion, and finally fusion with lysosomes to form autophagolysosomes (or namely amphysomes). Initiation of autophagy is mediated by a membrane nucleation event, which requires enrollment of the ULK1 complex and other molecules including FIP200, ATG13, ATG9, ATG6 (Beclin1), and also ATG5-ATG12-ATG16 complex (Fig. 1). In this scenario, ULK1 and ATG13 interact with ATG17 to form a ULK1-ATG13-ATG17 complex, which initiates autophagosome development in presence of ATG9. Simultaneously, PI3K-III nucleation complex containing Beclin1, VPS14, VPS35, and ATG14 promotes autophagy flux. Indeed, once autophagy is initiated, ATG14 backlog on the ER-mitochondrion interaction surface and promotes the nucleation step. In this situation, ATG4 catalyzes the formation of LC3-I from LC3, whereas ATG7 and ATG3 form LC3-II from LC3-I. In this conjugation system, the phosphatidylethanolamine (PE) conjugates to LC3-II and phagophor is being maturated to autoghagosome. In the expansion step, the PE-conjugated LC3-II located on both sides of the membrane of autophagosome. In the final step, LC3-II molecules are detached from the cytoplasmic side of the membrane and autophagosomes fuse with lysosomes to form autolysosomes where cargos are hydrolyzed (Fig. 1). The intracellular trafficking of autophagic vesicles is complex and mediated by different molecules such as microtubules, LAMP1/2, Rab7, soluble NEM-sensitive factor (NSF) attachment protein receptor (SNARE) proteins, VPS, and endosomal sorting complexes required for transport (ESCRT) complex.

## Exosome biogenesis

**Fig. 2:**
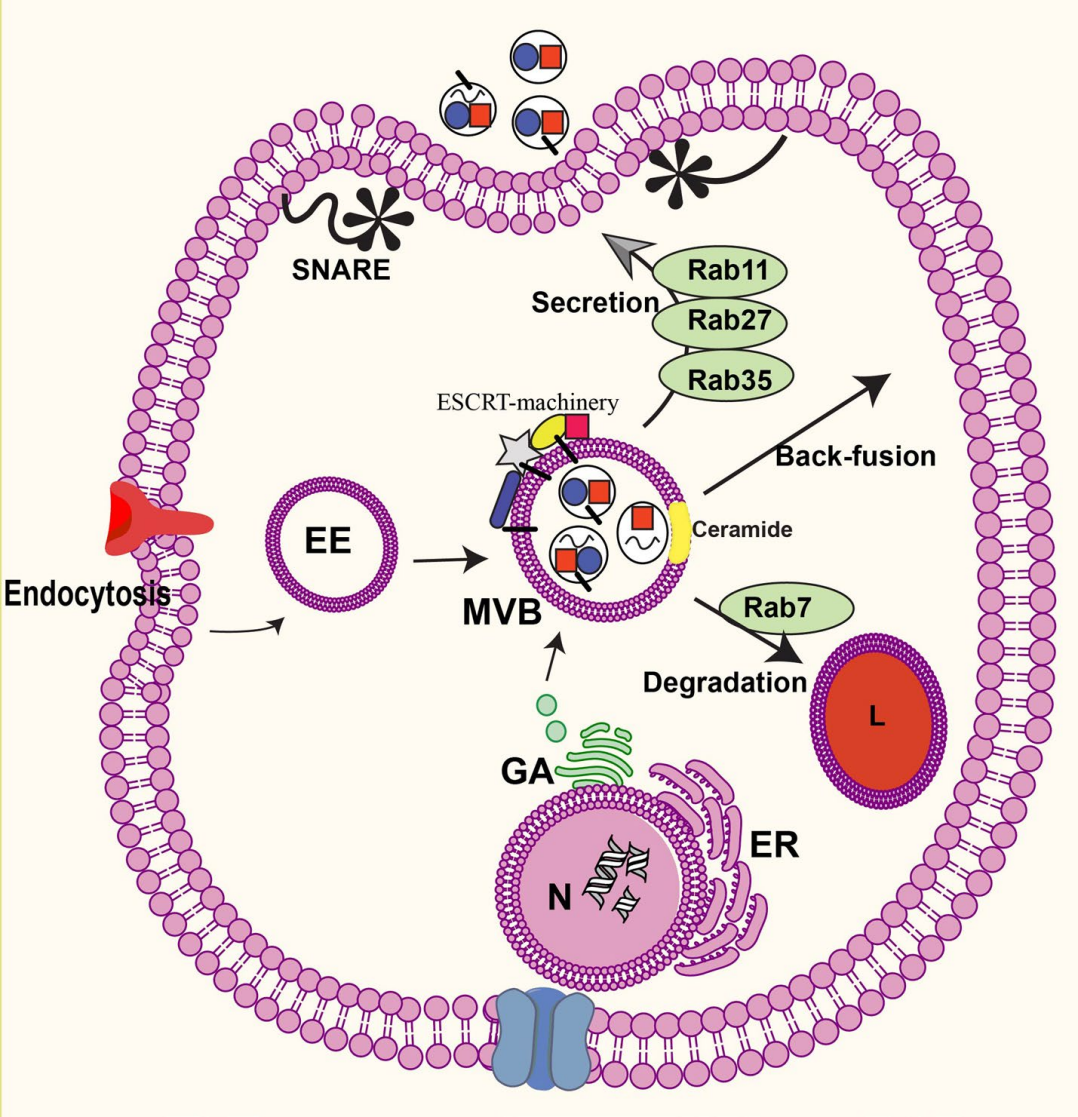
The exosome pathway. These small vesicles (30–120 nm) of endocytic origin are formed by inside budding of the membrane of late endosomes, generating multivesicular bodies (MVBs), and are released into the extracellular matrix by fusion of the MVBs with the plasma membrane (secretion pathway). Alternatively, MVBs may fuse with lysosomes for hydrolysis of exosomes (degradation pathway) or back-fuse with the plasma membrane for recycling such molecules (back-fusion pathway). Exosome cargo may consist of endocytosis, Golgi apparatus, and cytoplasm. Various molecules contribute to biogenesis of exosomes, including the ESCRT machinery, tetraspanins and lipids (ceramide). It is still unclear whether these mechanisms simultaneously generate the same MVB or not. Various Rab-GTPase proteins (Rab7, Rab11, Rab27a,-b, and Rab35) are involved in the intracellular trafficking of MVBs/exosome. Furthermore, SNAREs have been proposed to facilitate the fusion of MVBs with the plasma membrane. EE: early endosome; ER: endoplasmic reticulum; GA: Golgi apparatus; L: lysosome; N: nucleus

The extracellular vesicles (EVs) term refers to nano-sized vesicles releasing from the most mammalian cells into the extracellular environment [23]. Three types of EVs have been classified according to their origin and size; the endosomal-derived one is exosomes with 30–120 nm in diameter [24] (Fig. 2). These vesicles contain a different form of biomolecules including nucleic acids, proteins, lipids, and carbohydrates that play pivotal roles in the intracellular communication [25]. Exosomes initially are generated from endosomal compartments and share different networks of relations with endocytosis, lysosomal degradation and autophagy. In the endosomal pathway, trapped cargo or molecules placed on the plasma membrane (PM) are packed into early endosomes which finally are either recycled to the PM or sorted into late endosomes also called MVB. MVBs cargo may be directed into intraluminal vesicles (ILVs) for consequent secretion into extracellular milieu or directed to lysosomes for degradation [24, 26] (Fig. 2). Origin of exosomes is endosomal membrane, indeed, invagination of the MVB membrane forms ILVs inside MVB which are finally released outside of the cell when MVB combines with the PM [24, 26]. The stress and diseases condition may participate in inducing exosomes biogenesis, which we have recently described [27, 28].

Several studies have been published on exosome biogenesis [29, 30]. These studies demonstrated that different synchronized mechanisms contribute to exosome biogenesis which involves ESCRT-dependent machinery and ESCRT-independent machinery [29, 30] (Fig. 2). The ESCRT machinery has been demonstrated to composed of four complexes (ESCRT 0, ESCRT I, ESCRT II, and ESCRT III) and auxiliary molecules placed on the MVBs membrane which mediate the development of ILVs inside the MVBs and direct the ubiquitinated proteins into ILVs in presence of ATPase enzyme [24, 31]. The cascade of interaction among ESCRT subunits and accessory molecules leads to ILVs generation. Increasing evidence showed that exosomes from;diverse cell origin contain the common markers, for example, CD63, CD81, CD82, CD9, hepatocyte growth factor-regulated tyrosine kinase substrate (HRS), apoptosis-linked gene 2-interacting protein X (ALIX) [32, 33].

In the ESCRT-independent machinery, subunits other than ESCRT-dependent machinery including several lipids, tetraspanins, proteins, and microdomains (membrane typology) participate in MVB’s membrane inward budding and exosomes sorting [24]. In this scenario, for instance, ceramide a waxy lipid molecule plays a pivotal role in biogenesis of ILVs from MVBs of glial cells [34]. As a matter of fact, proteolipid proteins (PLP) are sorted into ILVs in lack of the ESCRT machinery subunits by raft-based microdomains that richly associated with sphingolipids, from which ceramides are made by the activity of an enzyme known as sphingomyelinases. Ceramide stimulates assembly of the microdomains and prompts ILVs generation [34] (Fig. 2). In parallel, other molecules such as CD63, CD9, CD82, and phospholipase D2 have been reported to play essential roles in the development of MVBs in different cells [35–37]. Collectively, ESCRT-dependent or independent machineries are involved in exosome biogenesis; however, it is indistinct whether both types of machinery work inside a cell concurrently in a synergy or independent manner, and whether several types of exosomes, and their cargo are made via collaboration of such distinct machineries in these pathways or not [24, 38]. Moreover, biomolecules exported from Golgi apparatus, engulfed from endocytosis pathway and/or from autophagosomes participate in exosome biogenesis and loading processes [39].

Along with crosstalk with autophagy, the MVBs also share collaborations with lysosomal degradation pathway. Outlined in the literature [24, 40], the mature MVBs may be degraded by the fusion with lysosomes or contribute to the recycling of biomolecules by back-fusion with the PM. In a well-known pathway, MVBs directly fuse with the PM and secrete ILVs as exosomes into the extracellular matrix.

Other molecules including Rab-GTPase family facilitates trafficking of MVBs/exosomes inside cells [41]. For example, in the degradation pathway, Rab7 mediates trafficking of MVBs to lysosomes for hydrolysis of MVBs cargo to adjust energy balance. Rab4 and Rab11 facilitate back-fusion of MVBs with the PM and decoration of the PM with surface molecules such as the major histocompatibility complex (MHC) and receptors. Whereas, Rab11, Rab27, and Rab35 are involved in MVBs fusion with the PM to release exosomes into extracellular milieu [42]. It has been suggested that SNAREs associated with the Rab proteins contribute to the fusion events of MVBs with the PM [39].

## Interaction between autophagy and exosome biogenesis

### Via molecular system

**Fig. 3:**
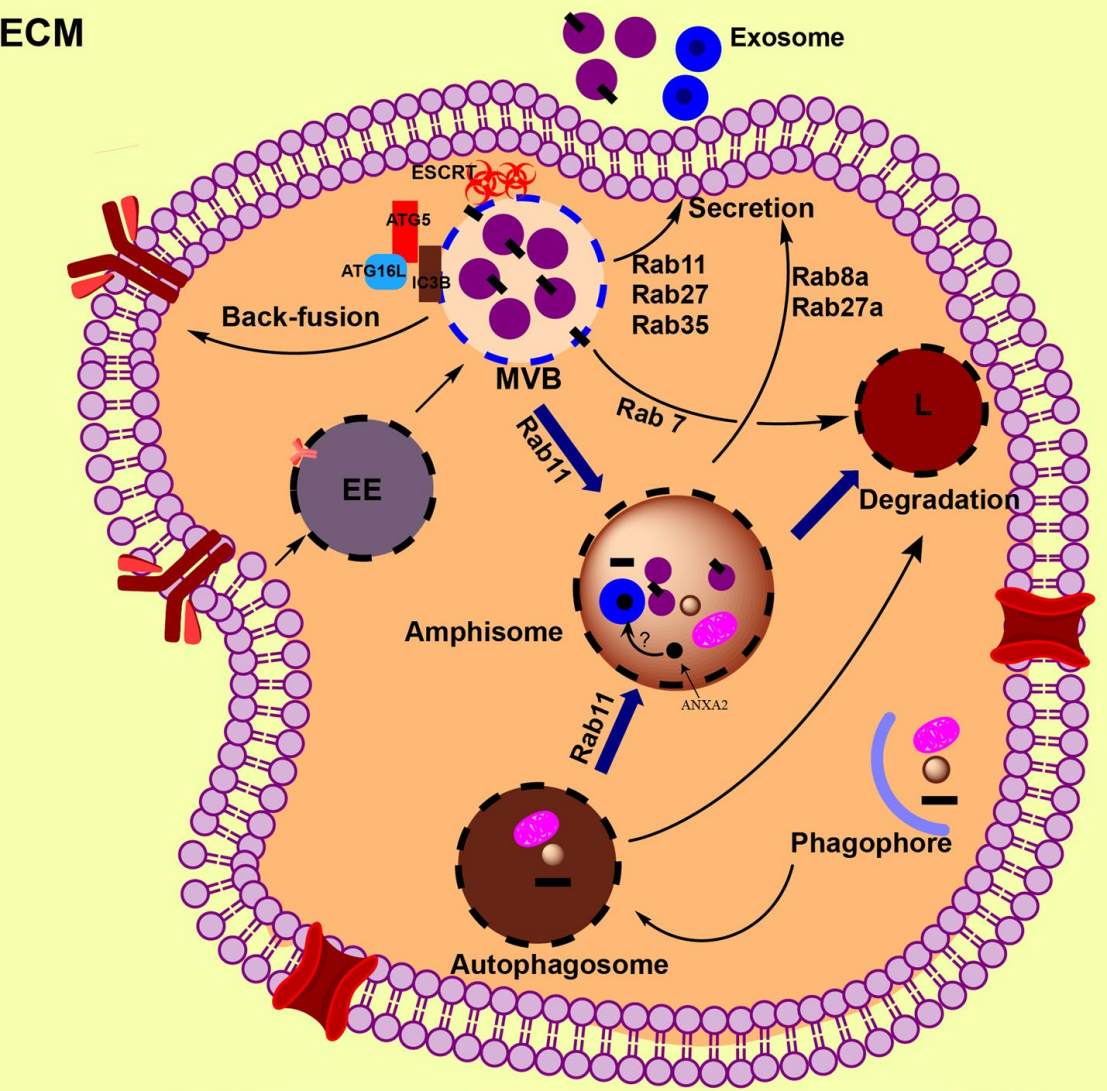
Interaction between exosome biogenesis and autophagy. Beside link at molecular level, exosome biogenesis and autophagy pathways meet each other by the hybrid-vesicles known amphisomes. In this link, the common molecules including Rab11, Rab8a, and Rab27 mediate the trafficking of vesicles between exosome and autophagy pathways. Different autophagic-related proteins (ATG5, ATG16L1, and LC3β) located on the multivesicular bodies (MVBs) membrane, facilitate exosome biogenesis, distributing via exosome into extracellular matrix. Amphisomes are crossbreed vesicles of MVBs and autophagosome that may fuse with lysosomes or with the plasma membrane. It was proposed that biomolecules such as annexin A2 (ANXA2) are sorted into exosomes at amphisomes lumen. Amphisomes, similar to MVBs, can fuse with lysosomes or with the plasma membrane, however, molecular mechanisms involved in amphiboles fate is still remains unknown. EE: early endosome; L: lysosome

Interaction between exosome biogenesis and autophagy pathway has been reported [43] (Fig. 3). Recent evidence suggests that the common molecules contribute to generating exosomes and autophagy flux. For example, SNARE proteins not only facilitate the fusion of MVBs with the PM but also mediate autophagy membrane fusion. In this regard, Nair et al. showed that maturation of autophagosome requires membrane fusion, which is depended on the activity of SNARE family proteins such as VAMP7, syntaxin 7, and syntaxin 8 [44]. VAMP7 plays a pivotal role in exosome secretion and is a key molecule for autophagy flux. Thus, SNAREs activity could represent the interaction between autophagosome/exosome biogenesis [45]. Furthermore, Rab11 protein, a MVB associated protein, has been reported to act as a platform for ATG proteins during the assembly of autophagosomes [46]. Previously, ALIX has been shown to associate with exosomal cargo, thus, it was suggested that ALIX association mediates discrepancy between lysosomal degradation and exosomal secretory pathways [47, 48]. In addition, ALIX inhibition experiments showed a fundamental decrease in autophagy, signifying a crosslink arrangement between exosome biogenesis and autophagy pathway [49]. ATG12–ATG3 complex has ability to regulate MVB’s shape, distribution of late endosomes, and eventually exosome biogenesis [49]. Interestingly, inhibition of the ATG12–ATG3 complex or ALIX did not inhibit starvation-induced autophagy that showing the contribution of the different overriding complexes in stress-induced and basal autophagy [50].

Growing evidence suggests autophagy-related proteins contribute to exosome biogenesis in normal and pathological conditions [49, 51]. For example, in pancreatic tumor cells, autophagy-related proteins including G alpha interacting protein (GAIP) and C-terminus (GIPC) induced exosome secretion via metabolic pathways [43]. Guo et al. reported that ATG16L1 and ATG5 play the pivotal roles in exosome biogenesis [51]. They showed that exosome biogenesis and exosomal lipidated LC3β secretion were significantly inhibited in breast cancer cells which are deficient in ATG5 and ATG16L1. ATG5 mediates detachment of V 1/V 0–ATPase (vacuolar proton pumps) from MVBs which, in turn, inhibits acidic environment of the MVBs lumen and directs MVBs to the PM instead of lysosomes. These results added further information that MVB’s lumen pH is a determinant signal of exosomes fate [51]. Little is known about the role of LC3β in exosome biogenesis and it is not clear how this molecule participates in exosome secretion. This molecule is found on the inside face of ILVs, proposing the LAP-like lipidation mechanism on the MVB’s membrane or at the membrane invagination border that subsequently produces ILVs. As a result, secretion of the LC3B containing exosomes suggests the involvement of the LAP-like mechanism in producing of non-degradative ILVs [51]. It is proposed that ATG16L1 and ATG5 shield exosomes from degradation pathway and direct them into the secretory pathway. Other autophagic components such as ATG12–ATG3 complex, which facilitates LC3β conjugation, contributes to exosome biogenesis via interaction with ALIX, a protein that interacts with ESCRT machinery for producing ILVs [49].

In *Drosophila*, it has been confirmed that ATG9 facilities the generation of ILVs in the endosomal compartments. Indeed, inhibition of ATG9 induced perturbation in autophagy flux and reduced amount of the ILVs in autolysosomes and amphisomes, however, it was not determined whether these ILVs were released as exosomes or not [52]. Furthermore, class III PI3K complex shares a pivotal role in exosome secretory and autophagy pathways.

In mammalian, this complex is made up of VPS34, Beclin-1, p150, and several supplementary molecules that are involved in autophagy and endocytosis processes. PI3K is necessary for producing PI (3) P molecules, which facilitates membrane trafficking in endocytosis and autophagy processes. In this regard, the interaction of ATG14L with PI3K complex controls autophagosome maturity, while UVRAG involvement mediates endosome maturation indicating the determinative role of this complex [53]. Additionally, the PI3K complex associated with Run domain Beclin-1-interacting and cysteine-rich domain-containing protein (Rubicon) participate in LC3-based phagocytosis [54] and block both endocytosis and autophagy [55]. In human chronic myeloid leukemia (CML) cells, it was reported that the distribution of the PI3K complex reduced both autophagy and exosome biogenesis [56].

### Via vesicular system

Interaction between exosome secretion and autophagy via the vesicular system has been shown by the biogenesis of amphisome inside cells (Fig. 3). Amphisomes, hybrid vesicles, may illustrate the indication of crosslink between exosome biogenesis and autophagy pathways [57]. These vesicles are produced through hybridization of MVBs and autophagosomes, which finally combine with lysosomes for hydrolysis of cargos such as ILVs or fuse with the PM for releasing ILVs [57] (Fig. 3). In a study by Fader and co-workers, rapamycin or starvation treatment supported autophagy and MVB-autophagosome combination and inhibited exosome secretion in K562 cell line [58], which suggesting cells challenge against energy imbalance. Interestingly, blockage of exosomes secretion alternatively leads to direct MVBs to the autophagy pathway. It has been proven that ISGylation of TSG101, an ESCRT-I complex protein, induced protein congestion and degradation and also diminished formation of MVBs and exosome in vitro and in a mouse model [59]. However, the block of lysosome-endosome fusion via inhibition of autophagy (by abafilomycin A1) improved exosome secretion, which indicated autophagy regulates the degradation of MVBs bearing ISGylation-induced aggregate by lysosomes [59]. Following CD63 knockout, it has been recently demonstrated that unusual endocytic vesicles degraded by autophagy, however, inhibition of autophagy fairly increased exosome biogenesis [60]. These results highlighted the key role of CD63 in synchronizing endosomal and autophagic pathways.

In intestinal goblet cells, LC3β is associated with the endosomal molecules including EEA1, Rab11, and RAB7 on amphisome-like structures, which correlated with the generation of ROS that regulates the secretion of mucin granules [61]. Correspondingly, in lung epithelial cells, amphisomes mediate generation of exosomes containing annexin A2 (ANXA2) exosome [62]. Indeed, in these cells, IFN-γ induced autophagy and accumulation of ANXA2, CD63, and LC3β inside amphisomes, and RAB11 and RAB27A mediated fusion of amphisomes with the PM [62]. Noteworthy, this secretion of cargo is different from the exosome secretion pathway. Indeed, secretion of IL-1β via autophagy flux is related on MVBs/exosome biogenesis [63] but autophagosome–lysosome combination dose not related to MVBs [64], demonstrating that LC3β-positive IL-1β containing compartments fuse with the PM. Besides, RAB8A facilities the secretion of the IFN-γ-induced ANXA2 containing exosomes [24] and secretion of autophagy-dependent IL-1β [65].

Collectively, exosome and autophagy pathways synchronize the intracellular removal process, so each pathway may initiate in deficiency of the other one alternatively. Degradable MVBs may be introduced to autophagy pathway, and malfunctioning in autophagy may direct MVBs to the PM and releasing exosomes [66]. Furthermore, it seems that there is the interplay between autophagy flux and cellular senescence, so increased senescence-associated EVs may be related to the insufficient autophagy status of senescent cells [67]. It is possible that these mechanisms may contribute to the pathogenesis of ageing diseases [68]. In addition, both processes may work together to shield cell from stressors [69]. Due to diversity in used cell types and multipart in endosomal system, further inquiry is necessary to delineate the possible any more networks between these pathways.

## Modulation of autophagy via exosome vice versa

As mentioned, normal autophagy flux is essential for maintaining cell homeostasis, while the excess autophagy causes cell death, indicating that autophagy has both the protective and detrimental functions in the pathological settings [13]. Jiang et al. found that inhibition of autophagy contributes to improving ischemia/reperfusion (I/R) injury in an animal model. Molecular experiments showed that mesenchymal stem cells (MSCs)-derived exosomes reduced LC3-II/I ratio and autophagosome formation, whereas up-regulated p62 in heart tissue [70]. On the contrary, Jin and co-workers declared that exosomes from adipose-derived stem cells (ADSCs) improved diabetic nephropathy through induction of autophagy flux and reducing apoptosis rate in podocytes [71]. Fujita et al. reported that exosomes from cigarette smoke-induced bronchial epithelial cells result in airway fibrosis by autophagy regulation via the exosomal miR-210 [72]. Exosomes-related miRNAs (i.e. miR-19b, miR-20a/b, miR-21, miR-30a, miR-33, miR-125b, miR-130a, miR-214, miR-221/222, miRNA-223, miRNA-302a, and miR-758) have been suggested to regulate autophagy flux via modulation of PI3K-Akt-mTOR and AMPK-mTOR signaling and downstream autophagic molecules including ATG or Beclin1, P62, and ULK1; which consequently increased expression of ABCA1 and cholesterol efflux in the atherosclerosis and cardiovascular diseases [73]. It has been revealed that tumor-derived exosomes play a major role in different stages of tumor progression of all cancer types. Regarding the gastric cancer cell-derived exosomes, they could regulate pro-tumor activation (polarization) of neutrophils via autophagy as well as HMGB1/TLR4/NF-κB signaling pathway induction which promoted proliferation and migration of gastric cancer cells [74]. There is increasing evidence that modulation of autophagy may cause alternation in exosomes loading/secretory pathway. For example, autophagy inhibition increased synuclein alpha (SNCA) secretion by neuronal cells which consequently resulted in the spreading of SNCA in brain tissue [75]. SNCA, an important cytosolic protein, plays a critical role in restricting of synaptic vesicles. It reduces release of some neurotransmitters such as dopamine transporter, parkin (ligase), tau protein and beta amyloid (β-amyloid) [76–81]. Therefore, autophagy may affect exosomes cargo, secretion, and also the formation of hybrid autophagosome-exosome vesicles [82]. Of note, α-synuclein widely considered to be the most important factor in Parkinson’s disease development. The previous study demonstrated that following autophagy blockage by ATG5 silence, distribution of α-synuclein increased by the exosomes and toxicity in human neurons considerably diminished [83]. Similarly, it is worth to note that over-expression of tubulin polymerization-promoting protein (TPPP/p25) caused an increase in exophagy rate (exocytosis of autophagic components) of α-synuclein, indicating unconventional secretion of α-synuclein following an inhibition in autophagosomes/lysosomal pathway [84]. Secretory carrier membrane protein 5 (SCAMP5) is believed to mediate α-synuclein propagation under the stress conditions by modulating autophagy flux and exosome secretion [85]. Regarding the infected hepatocytes by hepatitis C virus (HCV), a leading cause of liver malignancies, it has been reported that inhibition of autophagy was a protective approach to increase survival rate of infected cells following the suppression of innate immune response. In another context, knockdown of autophagy-related genes (BCN1 and ATG7) by up-regulation of BST-2 gene could inhibit the HCV sorting into exosomes and subsequently suppressed extracellular distribution of HCV [86]. Sahu et al. documented that the knockdown of ATG7 resulted in an increased level of GAPDH in the exosome contents [87]. Miao et al. reported that rapamycin (an autophagy inducer) treatment decrease exosome secretion, but rapamycin in conjugation with bafilomycin A1 (an autophagy inhibitor) led to an incredible release of exosomes [88]. The intracellular effectors and pathways suggest the possible coordination between autophagy and exosome biogenesis [15], which serves as a tool for cells to response against various stress conditions. However, the stimulatory or inhibitory effects are varying due to the pre-conditions and origin of cells.

## Role of mesenchymal stem cell-derived exosomes in autophagy flux

Stem cell-derived exosomes exhibit potentially beneficial effects through modulation of autophagy flux in target cells [89]. Several attempts have been made to investigate the pivotal roles of MSCs-derived exosomes in several diseases [27, 90]. These vesicles have been identified to modulate autophagy flux of target cells and contribute to attenuate adverse condition of diseases. For instance, the protective role of MSC-derived exosomes against myocardial infarction is mediated by up-regulation of ATG13 [91]. Further scrutiny confirmed that 3-methyladenine, an autophagy inhibitor, inhibited the therapeutic effect of exosomes in an I/R model [91]. Additionally, exosome secreted from ADSCs considered as a therapeutic tool in diabetic nephropathy. In this regard, miR-486 cargo of these exosomes could be able to ameliorate the urine profiles and high blood glucose level through suppression apoptosis and induction of autophagy in podocytes [92, 93].

A controversy research reported that exosomes from human MSCs had potential to attenuate I/R injury by inhibition of autophagy flux through up-regulation of mTORC1/p-4eBP1 [94]. Exosomes from pigment epithelium-derived factor (PEDF)–over-expressing MSCs have been shown to contribute to neuroprotection through inducing autophagy, which attenuated cerebral I/R injury [95]. Previous studies have emphasized the therapeutic capacity of MSC-derived exosomes on the progression of cisplatin-induced acute nephrotoxicity; and revealed that these particles were capable of reducing apoptosis biomarkers and inflammatory-related cytokines following over-expression of 14-3-3ζ protein and interaction of it with ATG16L [96, 97]. Exosomes obtained from MSCs over-expressing miRNA-181-5p up-regulated expression of Beclin-1 (autophagic protein) and decreased expression of Bcl-2 (anti-apoptotic protein) in mouse hepatic stellate cells, resulting in augmented autophagy and apoptosis in fibrotic livers. However, these exosomes considerably inhibited pro-fibrotic genes including α-SMA, collagen I, fibronectin, and vimentin hepatic stellate cells, which inhibited CCl4-induced liver fibrosis in a mouse model [98].

More recently, it was demonstrated that MSCs-derived exosomes effectively protected hepatocytes against D-galactosamine and lipopolysaccharide (D-GalN/LPS)-induced damage via increasing levels of autophagic related proteins (LC3B and Becin-1) and suppression of pro-apoptotic proteins [99]. The similar result were obtained in a rat model of spinal cord injury where authors showed that neural stem cell-derived exosomes suppressed apoptosis and neuro-inflammation, whereas induced autophagy [93].

## Exosomal secretion of autophagic regulators

As mentioned above, exosomes contain several biologically active materials including nucleic acids, proteins, lipids, and carbohydrates [29] that deliver them to target cells. Exosomes considered to be the important tool in the induction of autophagy flux in target cells through transferring autophagic activator or/and autophagy-related molecules. Autophagy was activated in recipient cells after internalization of exosomes. For instance, exosomes from breast cancer cells were capable of inducing autophagy flux in recipient breast epithelial cells [97]. Even though the scientists are expected to discover the autophagic cargo of EVs, there is still not sufficient information about them.

Growing evidence indicates EVs derived from MSCs contain several mRNAs of autophagy-related proteins including Beclin-1, LC3, and ATG7, which increase the autophagy flux in hematopoietic stem cells [100]. Confirmed that, upon exosomes uptake, human breast epithelial cells produce ROS, which contributes to the increasing of autophagy flux [97]. For that reason, it is reasonable that the arrival materials potentially facilitate intracellular autophagy triggered by exosomes.

Besides the autophagic component, there is evidence that specific miRNA cargo of exosomes can affect the dynamic of autophagy in target cells. It was revealed that ionizing radiated astrocytes in brain produce exosomes enriched with miRNA-7, which induces autophagy in lung cells through targeting Bcl-2 in vitro and in vivo model [101]. Similarly, in a study by Song et al. it was found that exosomes transferring miR-7-5p from γ -irradiated lung epithelial cells induced autophagy in target cells [102]. Ying and co-workers showed that exosomes purified from genetically modified MSCs transfer miR-181-5p to HST‐T6 cells, mouse hepatic stellate cells, and to CCl4‐induced liver fibrosis mouse model which induced autophagy and improved liver injury [98]. In addition, previous studies demonstrated that miRNA-30a cargo of cardiomyocytes exosomes and miR-30d-5p cargo of exosomes of brain cells up-regulated autophagy in the recipient cells [103, 104]. Other exosomal miRNAs such as miRNA-221/222 has been shown to down-regulate autophagy in endothelial cells [105]. It was demonstrated that human MSCs-derived exosomes contain 14-3-3ζ proteins that induced autophagy in HK-2 cells in vitro. Exosomes-mediated autophagy inside HK-2 cells was reduced after inhibition of the 14-3-3ζ gene in human MSCs [106]. These findings provide novel information that exosomes may regulate autophagy through transferring the autophagic components or/and via the autophagic regulators.

## Signaling pathways involved in cross-regulation of exosomes-induced autophagy

**Fig. 4:**
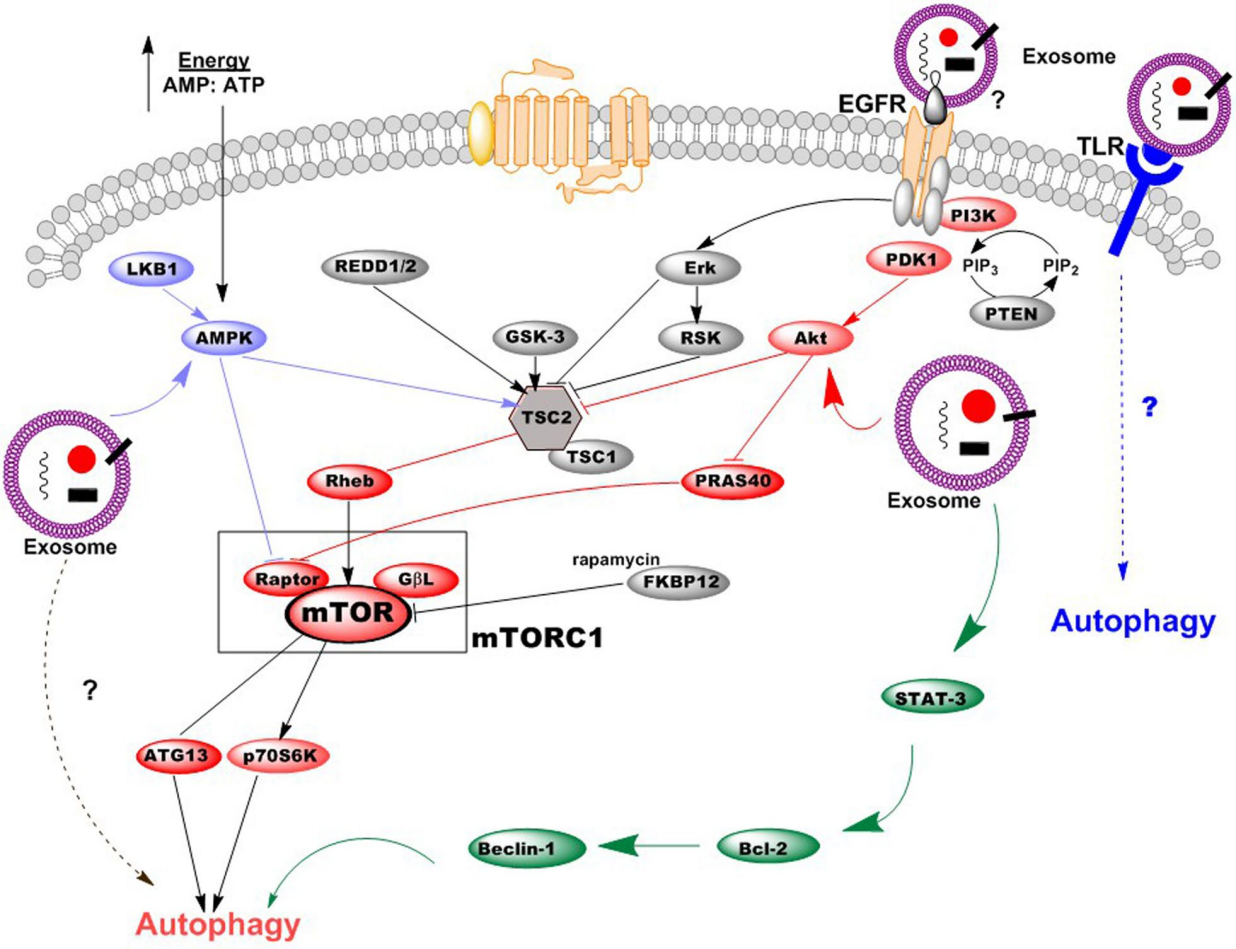
A schematic diagram of signaling pathways in exosome-mediated autophagy inside target cell. Different signaling pathways are involved in exosome-mediated autophagy. Exosomes may contribute to autophagy flux of target cells via directly delivering cargo into the cytoplasm or via interacting with such receptors as EGFR and TLR1/2/4/6 located on the plasma membrane. Other potential signaling pathways may be induced by exosomes. Red color for Akt/mTOR signaling pathway, Blue color for Toll-like receptor-ligand signal pathway, Light purple color for AMPK/mTOR signal pathway

A growing body of literature has investigated the biological responses of target cells received exosomes from the different sources [107, 108] (Fig. 4). As exosomes are distributed by much biological fluids, they can easily reach to target cells. Exosomes can regulate singling pathways of target cells through possible three ways including internalization, direct fusion, and ligand-receptor interaction (Fig. 4). These vesicles deliver several types of biomolecules to target cells by which may activate or/and inhibit different signaling pathways such as autophagic one. It was confirmed that exosomes secreted from breast cancer cells could connect with the normal human primary mammary epithelial cells (HMEC), and subsequently contributed to the tumorigenesis via increasing ROS generation and autophagy [109]. However, our knowledge is not more detailed due to numerous exosomes cargos and variety in cells/exosomes is investigated. Furthermore, because of technical limitation, the ways that exosomes used to affect target cells is not fully understood. In this section, we discuss the possible signaling pathways involved in the regulation of autophagy in target cells upon exosomes deliver.

### Cross-regulation by Akt/mTOR signaling pathway

Several singling pathways involved in autophagy flux (Fig. 4). Akt/mTOR signaling is an important component in the cellular system and plays the key roles in numerous cellular processes including survival, proliferation, growth, transcription, and angiogenesis [104]. It is confirmed that PI3K activates downstream signaling molecules such as AKT through converting phosphatidylinositol-4, 5-bisphosphate (PtdIns (4,5)P2) to phosphatidylinositol-3,4,5-triphosphate (PtdIns(3,4,5)P3) [110]. In addition, mTOR complex which is composed of two subunits (mTORC1 and mTORC2) regulates protein synthesis and mTORC1 subunit negatively regulates autophagy through phosphorylation of Atg 13 and inhibiting it from association with the ULK1 kinase complex [111]. In this regard, it has been proven that exosomes from MSCs potentially up-regulated the expression of LC3-II and Beclin-1 in the renal tissue of diabetic nephropathy (DN) mice through the activation of the mTOR signaling pathway, which behind the development of DN in mice. Moreover, the protective function of exosomes was proven in biochemistrical and histological analysis [92]. As a note, the pivotal role of mTOR and its downstream signaling molecule p70S6K in regulating autophagy has been confirmed [112]. PI3K/AKT/mTOR axis facilities growth and metabolism events of mammalians [113]. Recently, Xue et al. reported that Mitofusin2 was capable of inducing autophagy flux in pancreatic cancer cells via inhibiting the PI3K/AKT/mTOR pathway [114]. In a similar vein, it was demonstrated that treatment of lung cells with Perfluoroalkyl acid caused autophagy flux through suppressing the PI3K/AKT pathway [114]. Recent experiments indicated that the exosomes of MSCs have the potential to control autophagy through PI3K/AKT/mTOR pathway. For instance, MSCs-derived exosomes reduced oxidative stress and repressed myocardial remodeling in an I/R injury model by initiating PI3K/AKT signaling pathway [115], indicating modulating of autophagy through increasing ATP. In support, MSCs-derived exosomes have been found that to increase autophagy in H9C2 target cells. Concurrently, the transcripts of p-AKT/AKT and p-mTOR/mTOR were intensely decreased, while the p-AMPK/AMPK ratio augmented in the cells received exosomes. Thus, at least, these pathways mediated autophagy flux inside cells [109]. Surprisingly, exosomal miR-30a and miR-125b-5p which derived from transplanted MSCs represents the protective effect on I/R-induced injuries in cardiomyocytes by autophagy regulation through the Akt/AMPK/mTOR pathways both in vitro and in vivo studies [116–118]. It seems that activation of the PI3K/AKT/mTOR signaling pathway reduces excessive autophagy and rescues cells from death. In contrast, mTORC1 suppression triggers autophagy and eradicates intracellular toxicity. EFGR (epidermal growth factor receptor) has been confirmed to mediate multiple cellular processes including survival, growth, and differentiation of different types of cells [119]. EFGR in upstream situation regulates Akt/mTOR signaling pathway and play a pivotal role in autophagic pathway. In human bronchial epithelial cells, exosomes delivering miRNAs such as miRNA-7-5p induced autophagy through EGFR/Akt/mTOR pathway [102]. Under physiological conditions, EGFR-activated PI3K/AKT/mTOR signaling inhibits autophagy, while in tumors received therapies this pathway contribute to resistance via activation of autophagy. However, in tumors with resistance, EGFR targeting may provide us to overcome resistance [120]. As a result, this cross-regulation mediates exosomal-induced autophagy in target cells.

### Cross-regulation by STAT3/BCL-2/Beclin-1 signaling pathway

STAT3 has been proved an important negative regulator in autophagy process as it inhibits the protein kinase R [121]. A recent study showed that MSCs decreased the mRNA levels of STAT3 in human intrahepatic biliary epithelial cells, which resulted in autophagy activation [122]. A growing body of evidence has demonstrated the interaction between autophagy and apoptosis [114]. In this scenario, there is evidence that Bcl-2, an anti-apoptotic molecule, interacts with Beclin-1, which may result in inducing autophagy [123] (Fig. 4). Qu et al. showed that exosomes from MSCs suppressed expression of Bcl-2 and STAT5 in HSC-T6 cells. Further scrutiny revealed that these vesicles contain miR-181-5p that down-regulated Bcl-2 and STAT5 but up-regulated the Beclin-1 expression. Authors concluded that MSCs-derived exosomes induced autophagy via the STAT3/BCL-2/Beclin-1 axis [98].

### Cross-regulation by toll-like receptor-ligand signal pathway

Toll-like receptors (TLRs), membrane passing proteins, are abundantly expressed in immune cells and renal tissue, and considered as autophagy mediators. For instance, it was reported that TLR2 induced autophagy in acute kidney injury model made by cisplatin and improved the global renal tissue parameters [124]. Wen et al. found that TLR2 and TLR4 were up-regulated in hippocampus neurons of epilepsy mice. MiR-421-overexpression experiment showed that autophagy was inhibited in hippocampus neurons of epilepsy mice, indicating involvement of TLR/MYD88 signaling pathway [125]. Furthermore, there exist evidence that TLR2/1/CD14 signaling which activated by mycobacterial lipoprotein LpqH; induces antibacterial autophagy through cathelicidin and vitamin D receptor signaling [126]. Alvarez-Jimenez et al. demonstrated that exosomes from neutrophils infected with mycobacterium tuberculosis contain TLR2/6 ligands that induced autophagy flux in macrophages [127].

### Cross-regulation by AMPK/mTOR signal pathway

AMP-activated protein kinase (AMPK), a cellular energy regulator, contributes to autophagy flux. As mentioned previously, AMPK activates autophagy whereas mTOR inhibits autophagy. Xie et al. showed that hydrogen sulfide improved ischemic myocardium in murine model through AMPK-activated autophagy. In this regard, AMPK singling blocked mTOR activation that consequently resulted in autophagy flux [128]. Study of ezetimibe treatment showed that AMPK-TFEB pathway positively activated autophagy in cells [129]. Similarly, a study conducted by Zhao et al. showed that phosphorylation of AMPK promoted cytoprotection in myocytes following a hypoxia/reoxygenation injury [130]. In addition, AMPK/mTOR- mediated autophagy has been shown in an experiment that exosomes from MSCs increased autophagy in cardiomyocytes and diminished adverse effects of myocardial ischemia/reperfusion injury [109].

## Clinical translation potential of exosomes-mediated autophagy

Autophagy plays pivotal role in different pathological condition such as cancer and cardiovascular diseases (CVD) ([73] {Guo, 2013 #137)}. In tumor cells, both autophagy and exosome secretion are accelerated. Nutrient deprivation and hypoxia (which are present in the tumor environment) induce autophagy flux, which defends against inflammation and necrosis [131, 132]. In the case of cancer, autophagy is a bilabial process and in normal cells it contributes to inhibiting tumorigenesis, but in transformed cells, it promotes tumorigenesis. In tumor cells, autophagy also plays a dual role by supporting tumor growth and promoting tumor resistance to therapy [133]. Exosomes may also accelerate tumorigenesis through inducing autophagy in recipient cells. For instance, a study by Dutta et al. presented the novel mechanisms by which breast cancer cell derived exosomes manipulate normal human primary mammary epithelial cells (HMECs) to generate a tumor lenient microenvironment. In this context, they showed that exosomes from human breast cancer cells induce ROS production, phosphorylation of ATM, H2AX and Chk1 as well as induction of DNA damage repair (DDR) responses in HMECs, which eventually contributes to the increasing of autophagy flux and tumorigenesis [97]. Ma et al. found that exosomes from cisplatin-resistant non-small cell lung cancer (NSCLC) contain miRNA-425-3p that facilitate autophagy flux and induce cisplatin resistance in sensitive cells by targeting the AKT1/mTOR signaling pathway [134]. Similarly, exosomal miRNA-425-3p derived from cisplatin-resistant NSCLC cells declined sensitivity to cisplatin via targeting the AKT1/mTOR signaling pathway, which resulted in up-regulation of autophagic activity [135]. However, more recently Kulkarni et al. reported that exosome-mediated delivery of miRNA-30a sensitize cisplatin-resistant variant of oral squamous cancer cells via modulating Beclin1 and Bcl2, suggesting exosomes potential therapeutic role [136]. Tumor microenvironment is composed of the complex communication between cells, therefore, paracrine-mediated communication (such as exosomes) plays pivotal roles in signal transduction between neighboring and distant cells [137, 138]. It seems that exosomes can induce tumorigenesis through activation of autophagy in recipient cells, thus, targeting exosome-mediated autophagy may open new avenue to reduce tumor growth and resistance. In addition, the key role of autophagy in CVD has been well-studied in literature [139, 140]. Preclinical evidence suggests that autophagy is a double-edged sword in CVD, acting in either advantageous or maladaptive ways, depending on the context. In this regard, the autophagic machinery in cardiomyocytes and other cardiovascular cells has been suggested as a potential therapeutic target [139, 140]. MSCs-derived exosomes have been shown to improve CVD through regulating autophagy. Liu et al. found that exosomes from MSCs increased autophagy in cardiomyocytes through AMPK/mTOR pathway and reduced adverse effects of myocardial I/R injury [109]. Besides, MSCs-derived exosomes transfer miR-30a and miR-125b-5p, which participate in improving I/R-induced injuries in cardiomyocytes by modulating autophagy via the Akt/mTOR signaling pathways both in vitro and in vivo studies [116–118]. Therefore, MSCs derived exosomes have potential to modulate autophagy in cells of cardiovascular system, proposing clinical application in CVD treatment. Determining the role of exosome-mediated autophagy manipulation in clinical therapy will need approaches of assessing changes in autophagy in patients and their tumors/afflicted organs. Careful and exact assessment of autophagy with a focus on how to translate laboratory findings into related clinical therapies remains a vital feature of improving clinical outcomes in patients with diseases.

## Conclusions

Both exosome biogenesis and autophagy flux share crosslink not only at function but also at molecular signaling and vesicular levels. Crosstalk between these processes enables cells to respond appropriately against such stress conditions and mediates cell to cell communication. MSCs-derived exosomes demonstrate beneficial effects through modulating autophagy in vivo. Several autophagic molecules mediate exosome biogenesis, and autophagy and exosome biogenesis conjoin each other by the hybrid vesicles named amphisomes, which finally fuse with lysosomes or the plasma membrane. Exosomes transfer autophagic components and contribute to modulating autophagy in the target cells through different signaling pathways. More information on the interaction between exosomal and autophagic pathways would help us to establish a greater degree of accuracy on this matter for the treatment of various diseases such as cancer and CVD. Detailed mechanisms of this interaction is an intriguing one, some questions remain unresolved which could be usefully explored in further research. Dose cells produce different populations of MVBs and autophagosomes? How the fate of MVBs and autophagosomes differentially synchronized? Are autophagic proteins transferred by different exosomes? What are mechanisms involved in exosomal loading of autophagic component? Which are signaling mechanisms involved in modulating exosome-based autophagy in target cells?

## Data Availability

Data and materials are available upon request to corresponding author.

## Abbreviations

ABs: Apoptotic bodies
ADSCs: Adipose-derived stem cells
ALIX: Apoptosis-linked gene 2-interacting protein X
AMPK: AMP-activated protein kinase
ANXA2: Annexin A2
CMA: Chaperone-mediated autophagy
CML: Chronic myeloid leukemia
CVD: Cardiovascular diseases
DDR: DNA damage repair
D-GalN/LPS: d-Galactosamine and lipopolysaccharide
ECM: Extracellular matrix
ECs: Endothelial cells
EFGR: Epidermal growth factor receptor
ESCRT: Endosomal sorting complex required for transport
EVs: Extracellular vesicles
GAIP: G alpha interacting protein
GIPC: GAIP interacting protein C-terminus
HCV: Hepatitis C virus
HMECs: Human primary mammary epithelial cells
HRS: Hepatocyte growth factor-regulated tyrosine kinase substrate
HSC70: Heat shock cognate 71 kDa protein
ILVs: Intraluminal vesicles
ISEV: International society for extracellular vesicles
LAMP2A: Lysosomal-associated membrane protein type 2A
MHC: Major histocompatibility complex
MMPs: Matrix metalloproteinase
MSCs: Mesenchymal stem cells
mTORC1: mTOR complex 1
MVBs: Multivesicular bodies
NSCLC: Non-small cell lung cancer
nSMase2: Sphingomyelinase 2
PE: Phosphatidylethanolamine
PEDF: Pigment epithelium-derived factor
PLP: Proteolipid proteins
PM: Plasma membrane
PtdIns (4,5)P2: Phosphatidylinositol-4, 5-bisphosphate
PtdIns(3,4,5)P3: Phosphatidylinositol-3,4,5-triphosphate
ROS: Reactive oxygen species
SCAMP5: Secretory carrier membrane protein 5
SNAREs: Soluble NSF attachment protein receptors
SNCA: Synuclein alpha
TPPP/p25: Tubulin polymerization-promoting protein
TLR: Toll-like receptors
ULK1: Autophagy-initiating kinase Unc-51-like kinase 1
